# *PAX6* Haplotypes Are Associated with High Myopia in Han Chinese

**DOI:** 10.1371/journal.pone.0019587

**Published:** 2011-05-12

**Authors:** Bo Jiang, Maurice K. H. Yap, Kim Hung Leung, Po Wah Ng, Wai Yan Fung, Wai Wa Lam, Yang-shun Gu, Shea Ping Yip

**Affiliations:** 1 Department of Ophthalmology, The First Affiliated Hospital, Zhejiang University School of Medicine, Hangzhou, China; 2 Department of Health Technology and Informatics, The Hong Kong Polytechnic University, Hong Kong, Special Administrative Region, China; 3 School of Optometry, Centre for Myopia Research, The Hong Kong Polytechnic University, Hong Kong, Special Administrative Region, China; University of Swansea, United Kingdom

## Abstract

**Background:**

The paired box 6 (*PAX6*) gene is considered as a master gene for eye development. Linkage of myopia to the *PAX6* region on chromosome 11p13 was shown in several studies, but the results for association between myopia and *PAX6* were inconsistent so far.

**Methodology/Principal Findings:**

We genotyped 16 single nucleotide polymorphisms (SNPs) in the *PAX6* gene and its regulatory regions in an initial study for 300 high myopia cases and 300 controls (Group 1), and successfully replicated the positive results with another independent group of 299 high myopia cases and 299 controls (Group 2). Five SNPs were genotyped in the replication study. The spherical equivalent of subjects with high myopia was ≤−8.0 dioptres. The PLINK package was used for genetic data analysis. No association was found between each of the SNPs and high myopia. However, exhaustive sliding-window haplotype analysis highlighted an important role for rs12421026 because haplotypes containing this SNP were found to be associated with high myopia. The most significant results were given by the 4-SNP haplotype window consisting of rs2071754, rs3026393, rs1506 and rs12421026 (*P* = 3.54×10^−10^, 4.06×10^−11^ and 1.56×10^−18^ for Group 1, Group 2 and Combined Group, respectively) and the 3-SNP haplotype window composed of rs3026393, rs1506 and rs12421026 (*P* = 5.48×10^−10^, 7.93×10^−12^ and 6.28×10^−23^ for the three respective groups). The results remained significant after correction for multiple comparisons by permutations. The associated haplotyes found in a previous study were also successfully replicated in this study.

**Conclusions/Significance:**

*PAX6* haplotypes are associated with susceptibility to the development of high myopia in Chinese. The *PAX6* locus plays a role in high myopia.

## Introduction

Myopia is the most common human eye disorder in the world and has become a significant public health problem [1], [2]. High myopia, typically defined as a refractive error of −6.0 diopters (D) or worse, is associated with an increased risk of pathological ocular complications [3]. Myopia is regarded as a complex eye disease affected by both genetic and environmental factors as well as gene-environment interactions [4], [5]. The prevalence of myopia is significantly higher in Asian populations (∼70%) than in populations of European descent (<30%), especially in the younger generations in recent decades [1], [6]–[8]. The same also applies to the severity of myopia. This suggests that Asian populations are genetically more susceptible to particular environmental factors which cause myopia [9]. Family and twin studies support a high heritability of myopia and hence suggest a definite genetic basis for high myopia [4], [5], [10], [11]. Many myopia loci have been mapped by linkage studies.[4], [5] Quite a number of myopia susceptibility genes have also been identified by our group [12]–[15] and other groups, as has recently been reviewed [4], [5]. The majority of findings are conflicting, including those for the paired box 6 (*PAX6*, GeneID: 5080) gene on chromosome 11.

The *PAX6* gene is a member of the PAX family of transcription factors containing two DNA-binding motifs – the paired domain and the paired-type homeodomain. It is highly conserved, and essential for normal development of several organs including the brain, pancreas and the eye [16], [17]. *PAX6* has been considered as the master gene for eye development [18]. The correct dosage of *PAX6* is crucial for normal eye development: over-expression of *Pax6* in mice results in microphthalmia, retinal dysplasia and defective retinal ganglion cell axon guidance [19], whereas haplo-insufficiency in the mouse results in the phenotype of the small eye (*Pax6*+/−) or no eye (*Pax6*-/-) [20]. In humans, heterozygous *PAX6* mutations cause aniridia as well as other various congenital eye abnormalities [21]. Of note is that most mutations are truncating or loss-of-function and cause aniridia.

*PAX6* was first reported as a candidate gene for myopia in a genome-wide linkage scan for myopia susceptibility loci in a twin study, and was in fact directly below the highest peak at the 11p13 locus – the *MYP7* locus [22]. The same study and two other studies, however, did not found the association of *PAX6* single nucleotide polymorphisms (SNPs) and **common** myopia [22]–[24]. Intriguingly, three Chinese studies reported positive association between **different** *PAX6* polymorphisms and **high** myopia [14], [25], [26]. In addition, an Australian group found rare nonsense *PAX6* mutations in family members with high myopia [27]. It seems that *PAX6* is more likely a susceptibility gene for high myopia, rather than common myopia. Even for high myopia, the three positive association studies have however suggested three different polymorphisms [14], [25], [26], and the SNP rs667773 [25] could not be replicated in Han's study [14]. This warrants a larger-scale study to clarify the role of *PAX6* in myopia. The present case-control study aims to investigate the relationship between high myopia and polymorphisms in the *PAX6* gene and all known regions involved in the regulation of *PAX6* expression. *PAX6* regulatory elements have been identified in a large region extending from ∼15 kb upstream of exon 1 of the gene to a downstream gene known as elongation protein 4 homologue (*ELP4*; Gene ID: 26610) [28]–[34].

## Methods

### Subject and DNA samples

In an initial association study, 600 unrelated Southern Han Chinese subjects (**Group 1**) were recruited: 300 cases of high myopes with spherical equivalent (SE) ≤−8.00 D in both eyes, and 300 emmetropic controls with SE within ±1.00 D in both eyes. A follow-up replication study was performed to validate the results of the initial phase with a second cohort of 598 unrelated Han Chinese subjects (**Group 2**) with equal number of cases and controls. The same entry criteria were used for recruiting subjects of Group 1 and 2. This study was approved by the Human Subjects Ethics Subcommittee of the Hong Kong Polytechnic University, and adhered to the tenets of the Declaration of Helsinki. Signed, informed consents were obtained from all participants. All subjects were recruited from the Optometry Clinic of the Hong Kong Polytechnic University, and the complete ocular examination, collection of blood samples and DNA extraction were performed as described previously [15]. Below is a summary of the study subjects.

**Group 1** included 300 high myopia cases and 300 emmetropic control subjects (Table 1). For Group 1 cases, the mean age was 27.7 years (range, 15–48); mean SE was −10.03 D (range, −27.75 to −8.00); and mean axial length (AXL) was 27.72 mm (range, 24.98 to 31.66). For Group 1 controls, the mean age was 24.9 years (range, 17–46); mean SE was 0.04 D (range, −1.00 to 0.88); and mean axial length was 23.81 mm (range, 21.33 to 26.23). **Group 2** had 299 high myopia cases and 299 control subjects (Table 1). For Group 2 cases, the mean age was 34.0 years (range, 17–51); mean SE was −10.28 D (range, −25.15 to −8.00); and mean AXL was 27.64 mm (range, 24.80 to 31.81). For Group 2 controls, the mean age was 32.5 years (range 17–54); mean SE was 0.06 D (range, −0.93 to 1.00); and mean AXL was 23.67 mm (range, 21.45 to 25.91). The correlation of SE between right and left eyes was 0.97 in both groups. As expected, the partial correlation of SE with other ocular components was the best for axial length (Table 1).

**Table 1:** Clinical characteristics of study subjects.

	Group 1					Group 2			
Characteristic	Cases, n = 300		Controls, n = 300			Cases, n = 299		Controls, n = 299	
Age, mean (SD), yr	27.7	(6.9)	24.9	(6.1)		34.0	(9.1)	32.5	(9.7)
Female, No, (%)	217	(72.3%)	169	(56.3%)		195	(65.2%)	180	(60.2%)
SE, mean (SD), mm	−10.03	(3.70)	0.04	(0.39)		-10.28	(2.31)	0.06	(0.47)
AXL, mean (SD), mm	27.72	(1.11)	23.81	(0.79)		27.64	(1.13)	23.67	(0.79)
CP, mean (SD), D	44.08	(1.28)	43.37	(1.50)		44.01	(1.38)	43.73	(1.44)
ACD, mean (SD), mm	3.73	(0.29)	3.61	(0.28)		3.44	(0.33)	3.28	(0.33)
LT, mean (SD), mm	4.06	(0.57)	3.92	(0.44)		4.21	(0.45)	4.23	(0.48)
Partial correlation with SE, r (*P* value)									
AXL	−0.38	(<0.001)	−0.25	(<0.001)		−0.53	(<0.001)	−0.26	(<0.001)
CP	−0.11	(0.06)	−0.09	(0.13)		−0.13	(0.03)	0.006	(0.93)
ACD	0.18	(0.002)	−0.28	(0.003)		0.09	(0.16)	−0.17	(0.01)
LT	−0.03	(0.64)	0.07	(0.21)		−0.03	(0.68)	0.03	(0.68)

Abbreviations: ACD, anterior chamber depth; AXL, axial length; CP, corneal power; D, dioptre; LT, lens thickness; SE, spherical equivalent.

a The ocular data are the average measurements of both eyes.

### Selection and genotyping of SNPs

Tag SNPs were selected using Tagger [35] from a region of 324.6 kb (chr11: 31,484,873..31,809,434; NCBI B36 assembly) that encompassed the *PAX6* gene and its potential regulatory regions (20 kb upstream of *PAX6* and 282.16 kb downstream of *PAX6*). Note that the downstream regulatory region embraces the whole *ELP4* gene, which is transcribed in the opposite direction. Based on HapMap data for Han Chinese (release #24/phase II), Tagger used the following criteria for SNP selection: pairwise tagging algorithm, r^2^≥0.80 and minor allele frequency ≥0.10. Five additional SNPs were also included because simulation study has shown that, in case of positive association between the markers and the phenotype, strong linkage disequilibrium (LD) between markers does not necessarily guarantee correlated association test results [36]. These five SNPs (rs667773, rs3026390, rs2071754, rs1506 and rs12421026) had also been examined in at least one of the previous studies and, in particular, all except rs2071754 were found to be associated with high myopia in single-marker or haplotype analysis [14], [22]–[25].

Two different methods were used to genotype these SNPs: restriction fragment length polymorphisms or unlabelled probe melting analysis [37]. Details of primer sequences and reaction conditions are given in the online Appendix S1. The choice of methods depended on the logistic arrangement for instrument use in our core laboratory and the cost of the assays.

### Statistical analysis

High myopia was examined as a dichotomous trait. Subjects were classified as affected (cases) or unaffected (controls). PLINK (ver. 1.07; http://pngu.mgh.harvard.edu/~purcell/plink/index.shtml) was used for the analysis of genetic data: Hardy-Weinberg equilibrium (HWE) on unrelated subjects and association analysis [38]. Exact tests for HWE were performed for controls and cases separately. Haplotype analysis with sliding windows was also performed with PLINK, and multiple comparisons was corrected by generating empirical *P* (*P*_emp_) values based on 50,000 permutations. Haplotype blocks were constructed with Haploview (http://www.broadinstitute.org/haploview) only for the initial study with an algorithm known as the solid spine of linkage disequilibrium (SSLD) [39].

## Results

### Initial study – Group 1 subjects

**Figure 1:**
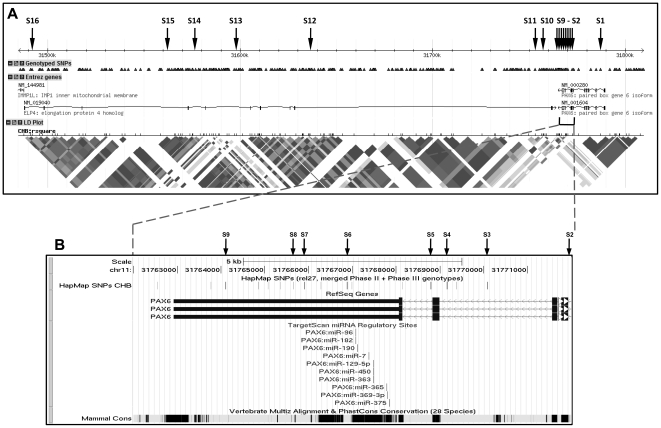
Distribution of 16 single nucleotide polymorphisms (SNPs) in the *PAX6* region. The *PAX6* region under study spans a genomic region of 324.6 kb (chr11: 31,484,873..31,809,434) together with different interesting features. Please refer to Table 1 for the rs numbers of the SNPs. (A) The top panel shows the physical positions (+ strand) of the 16 SNPs (S1 to S16, right to left) in the region under study, followed by positions of the SNPs genotyped by the HapMap project, the Entrez genes (*ELP4* and *PAX6*) and the linkage disequilibrium (LD) pattern (r^2^) for HapMap Han Chinese. Note the low LD region in the 3′ end of the *PAX6* gene. (Source: http://hapmap.ncbi.nlm.nih.gov/index.html.en) (B) A closer view of the 10-kb region in the 3′ end of the *PAX6* gene, the binding sites for ten micro-RNAs as predicted by the programme TargetScan, and the sequence conservation across 28 mammalian species. (Source: http://genome.ucsc.edu/cgi-bin/hgGateway).

Eleven tag SNPs were selected, and could capture the genetic information for a total of 105 SNPs in the indicated region (324.6 kb) at a mean r^2^ of 0.969. Five additional SNPs were selected as has been explained above. In total, 16 SNPs were examined, and are designated as S1 to S16 in sequential order from the 5′ end of the *PAX6* gene for the sake of easy reference (Figure 1A and Table 2). Half of the markers (S2 to S9) clustered in a low LD region of ∼8 kb at the 3′ end of *PAX6*.

**Table 2:** Summary statistics of *PAX6* SNPs in the initial study and the replication study: Genotype counts, Hardy-Weinberg equilibrium testing, minor allele frequencies and single-marker association analysis.^a^

	Allele		Genotype counts (11/12/22)		HWE (*P* value)		Minor allele freq		Association (best result)	
SNP	1	2	Cases	Controls	Cases	Controls	Cases	Controls	*P* value	Model
**Initial study – Group 1 subjects**										
rs3026354(S1)	T	C	144/129/27	159/115/26	0.8919	0.4718	0.3050	0.2783	0.2206	Dom
rs667773(S2)	C	T	191/102/7	181/110/9	0.1407	0.1242	0.1933	0.2133	0.3668	Add
rs3026390(S3)	A	G	103/146/51	96/146/58	1.0000	0.9065	0.4133	0.4367	0.4136	Allelic
rs2071754(S4)	G	A	101/141/58	89/144/67	0.4811	0.5621	0.4283	0.4633	0.2226	Allelic
rs3026393(S5)	T	G	89/163/48	81/161/58	0.0775	0.2013	0.4317	0.4617	0.2726	Add
rs1506(S6)	A	T	69/167/64	78/154/68	0.0643	0.7286	0.4917	0.4833	0.3929	Dom
rs12421026(S7)	G	A	95/162/43	85/158/57	0.0573	0.2970	0.4133	0.4533	0.1251	Rec
rs662702(S8)	G	A	195/94/11	181/107/21	1.0000	0.5019	0.1933	0.2183	0.2373	Dom
rs3026401(S9)	G	A	62/176/62	71/171/58	0.0038	0.0155	0.5000	0.4783	0.3764	Dom
rs7125966(S10)	G	C	96/166/49	89/147/55	0.3443	0.4129	0.4217	0.4433	0.4356	Add
rs2863231(S11)	T	C	109/142/48	107/147/46	0.9047	0.8095	0.3983	0.3983	0.8223	Rec
rs964112(S12)	G	T	80/159/61	90/144/66	0.2977	0.5623	0.4683	0.4600	0.3649	Dom
rs7947424(S13)	C	T	133/140/27	133/133/34	0.2918	1.0000	0.3233	0.3350	0.3443	Rec
rs11031423(S14)	T	C	192/99/9	188/97/15	0.4635	0.6037	0.1950	0.2117	0.2113	Rec
rs11031419(S15)	A	T	208/86/6	195/101/4	0.5266	0.0304	0.1633	0.1817	0.2584	Dom
rs509628(S16)	C	T	119/148/33	125/138/37	0.2109	1.0000	0.3567	0.3533	0.6110	Rec
**Replication study – Group 2 subjects**										
rs3026390(S3)	A	G	107/141/51	111/133/55	0.719	0.1875	0.4064	0.4064	0.6684	Rec
rs2071754(S4)	G	A	98/145/55	99/135/65	0.906	0.1593	0.4279	0.4431	0.317	Rec
rs3026393(S5)	T	G	106/143/50	99/138/62	0.905	0.2909	0.4064	0.4381	0.2085	Rec
rs1506(S6)	A	T	80/159/60	85/141/73	0.295	0.3544	0.4666	0.4799	0.2011	Rec
rs12421026(S7)	G	A	98/151/50	110/131/58	0.555	0.0959	0.4197	0.4130	0.2588	Geno

a The SNPs are arranged in sequential order from the 5′ end to the 3′ end of the sense strand of the *PAX6* gene, and are also designated as S1 to S16 for the sake of easy reference and discussion. The major allele is designated as “1” and minor allele as “2”; and the genotype counts are indicated as the counts of the genotypes 11, 12 and 22, respectively. HWE stands Hardy-Weinberg equilibrium. Single-marker association analysis is performed under five models for each SNP: genotypic (Geno), additive (Add; tested by Armitage trend test), dominant (Dom), recessive (Rec) and allelic models. However, only the best result and the corresponding model are shown for each SNP. Note that single-marker analysis did not show significant results for any of the 16 SNPs tested.

**Figure 2:**
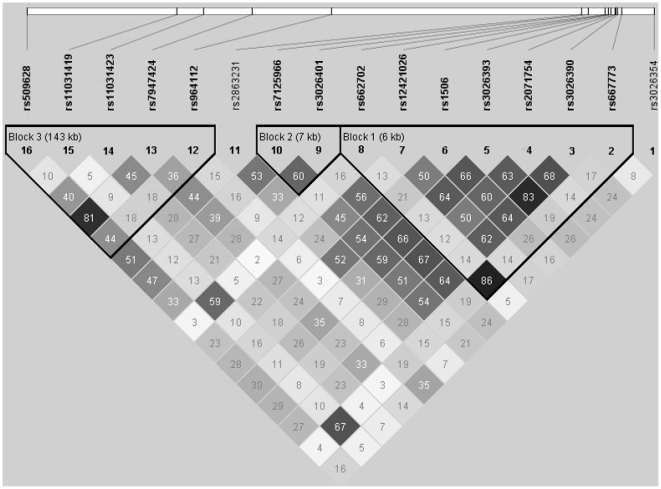
Linkage disequilibrium (LD) pattern across the 16 single nucleotide polymorphisms under study. LD measures are expressed as r^2^ in Group 1 subjects (cases and controls combined). The LD blocks are defined using the solid spine of LD algorithm of Haploview.

All SNPs were in HWE if a threshold *P* value of 0.01 was used, except rs3026401 (S9) for the case group (*P* = 0.0038) (Table 2). Two SNPs showed marginally significant *P* values for HWE testing of the control group: 0.0155 for rs3026401 (S9) and 0.0304 for rs509628 (S16). This is not unexpected since about 2 significant results could be obtained due to random chance with 32 comparisons at a significance level of 0.05. As such, these two SNPs were also included for subsequent data analysis. We identified three haplotype blocks across the 324.6-kb region under study with the SSLD algorithm of the Haploview package (Figure 2). However, the LD among the 16 SNPs under study was in general quite weak with a few exceptions.

There were more female subjects in the case group than in the control group (72.3% vs 56.3%, *P*<0.0001; Table 1). However, allele frequencies did not show any significant differences between female and male subjects in either group for all 16 SNPs. This justified the direct comparison of genotypes between the controls and cases without stratification by sex. Table 2 shows the distribution of genotypes and minor allele frequencies in both cases and controls. There were no significant differences between cases and controls of Group 1 under all five genetic modes tested (genotypic, additive, dominant, recessive and allelic).

We compared haplotype frequencies between cases and controls with adjustment for sex as a covariate to avoid its potential confounding. Instead of performing haplotype analysis within the LD blocks identified, exhaustive haplotype analyses were conducted using a sliding window strategy and examining all haplotypes of all possible sizes (numbers of SNPs per haplotype) (Table 3). There were a total of 136 sliding windows, 69 of which were significantly associated with high myopia (omnibus test *P*_emp_ <0.05). Above all, there was at least one window showing significant result among sliding windows of a given size. For sliding windows with size up to 10 SNPs per window, it was obvious that the omnibus test result was significant as long as rs12421026 (S7) was included in the window. The crucial importance of S7 was even more obvious if one considered the most significant result among the group of sliding windows of a fixed size for up to 6 SNPs per window. The relative importance of SNPs within a sliding window was less apparent when the size of the sliding windows increased beyond 10 SNPs per window. Most importantly, the window of S4-S5-S6-S7 (indicated as S4..S7 in Table 3) gave the most impressive *P*_emp_ value for the omnibus test. Table 4 shows the details of haplotype analysis for the most significant 3-SNP and 4-SNP windows.

**Table 3:** Summary of exhaustive haplotype analyses based on sex-adjusted omnibus tests for sliding windows of all possible sizes across 16 SNPs under study for subjects of Group 1.^a^

Sliding window (SW)			SW with omnibus test *P*_emp_ <0.05				Most significant result		
SNPs/SW	No. of SW		No. of SW	First SW	Last SW		SW	*P*_asym_	*P*_emp_
1	16		0	-	-		-	-	-
2	15		2	S6..S7	S7..S8		S6..S7	1.12E-08	2.00E-05
3	14		3	S5..S7	S7..S9		S5..S7	5.48E-10	2.00E-05
4	13		4	S4..S7	S7..S10		S4..S7	3.54E-10	2.00E-05
5	12		5	S3..S7	S7..S11		S3..S7	2.24E-08	2.00E-05
6	11		6	S2..S7	S7..S12		S2..S7	4.32E-09	2.00E-05
7	10		7	S1..S7	S7..S13		S2..S8	6.70E-08	2.00E-05
8	9		7	S1..S8	S7..S14		S2..S9	7.48E-08	2.00E-05
9	8		7	S1..S9	S7..S15		S7..S15	3.81E-06	4.00E-05
10	7		7	S1..S10	S7..S16		S4..S13	5.24E-08	2.00E-05
11	6		6	S1..S11	S6..S16		S4..S14	1.40E-06	2.00E-05
12	5		5	S1..S12	S5..S16		S2..S13	7.79E-07	2.00E-05
13	4		4	S1..S13	S4..S16		S3..S15	1.99E-06	2.00E-05
14	3		3	S1..S14	S3..S16		S3..S16	3.87E-06	4.00E-05
15	2		2	S1..S15	S2..S16		S2..S16	2.23E-05	3.00E-04
16	1		1	S1..S16	S1..S16		S1..S16	1.09E-03	2.02E-02

a Exhaustive haplotype analyses were performed using sliding windows (SW) of all possible sizes (i.e. number of SNPs per SW; 1 to 16 SNPs per SW) with PLINK. With logistic regression, a single case-control omnibus test of (H - 1) degrees of freedom was carried out for each sliding window to jointly assess the significance of the haplotype effects for this SW and adjusted for sex (as a covariate), where H is the number of haplotypes for the SW under consideration. Here, a single asymptotic *P* value (*P*_asym_) was produced for each SW. For a given window size, the test was performed for all possible windows of the same size, shifting one SNP at a time. There were a total of 136 windows (the sum of numbers in column 2), and multiple comparisons were corrected by running 50,000 permutations to obtain an empirical *P* value (*P*_emp_). The minimum *P* value that is achievable with 50,000 permutations is 2.00×10^−5^. The SW is indicated as Sa..Sb, where a is the first SNP and b the last SNP of the SW. For example, S4..S7 refers to the SW S4-S5-S6-S7. The three rightmost columns show the most significant results for each fixed-size SW. Note that, among all 136 windows tested, S4..S7 ranks the first and S5..S7 the second in providing evidence for association with high myopia.

**Table 4:** Details of sex-adjusted haplotype analysis for 3-SNP and 4-SNP windows showing the most significant results among the all possible sliding windows.^a^

		Group 1						Group 2						Combined				
Haplotypes		Cases	Controls	OR	*P*_asym_	*P*_emp_		Cases	Controls	OR	*P*_asym_	*P*_emp_		Cases	Controls	OR	*P*_asym_	*P*_emp_
rs3026393-rs1506-rs12421026 (S5-S6-S7)																		
OMNIBUS		–	–	–	5.48E-10	2.00E-05		–	–	–	7.93E-12*	2.00E-05		–	–	–	6.28E-23*	2.00E-05
TAG ( 111)		0.4244	0.4690	0.781	0.0511	0.9950		0.4202	0.4852	0.757	0.0187	0.1811		0.4185	0.4753	**0.767**	0.0021	0.0315
TAA ( 112)		0.0576	0.0062	**12.7**	6.40E-05	0.0045		0.1104	0.0114	**20.8**	3.60E-07	2.00E-05		0.0844	0.0096	**16.8**	6.87E-11	2.00E-05
TTG ( 121)		0.0837	0.0612	1.38	0.1660	1.0000		0.0403	0.0643	0.601	0.0666	05290		0.0617	0.0627	**0.989**	0.9490	1.0000
GTG ( 221)		0.0774	0.0088	**10.3**	1.98E-06	0.0001		0.1194	0.0141	**11.4**	2.15E-07	2.00E-05		0.0985	0.0122	**10.7**	2.22E-12	2.00E-05
GTA ( 222)		0.3434	0.4447	**0.618**	0.0002	0.0127		0.2838	0.4005	**0.592**	3.14E-05	0.00014		0.3105	0.4209	**0.606**	2.29E-08	2.00E-05
rs2071754-rs3026393-rs1506-rs12421026 (S4-S5-S6-S7)																		
OMNIBUS		–	–	–	3.54E-10*	2.00E-05		–	–	–	4.06E-11	2.00E-05		–	–	–	1.56E-18	2.00E-05
GTAG (1111)		0.4173	0.4637	0.752	0.0227	0.8958		0.4335	0.4864	0.773	0.0301	0.2292		0.4273	0.4739	**0.761**	0.0015	0.0217
GTAA (1112)		0.0518	0.0065	**10.7**	1.62E-04	0.0118		0.1123	0.0114	**19.4**	4.25E-05	2.00E-05		0.0832	0.0095	**15.9**	1.88E-10	2.00E-05
GTTG (1121)		0.0628	0.0417	1.49	0.1620	1.0000		0.0207	0.0371	0.529	0.0920	0.6592		0.0419	0.0391	1.04	0.8680	1.0000
AGTG (2221)		0.0654	0.0073	**10.7**	1.76E-05	0.0011		0.1199	0.0123	**13.8**	3.88E-07	2.00E-05		0.0939	0.0104	**11.8**	2.84E-11	2.00E-05
AGTA (2222)		0.3179	0.4274	**0.583**	2.62E-05	0.0118		0.2912	0.4014	**0.600**	4.95E-05	0.0003		0.3058	0.4132	**0.594**	2.90E-09	2.00E-05

a Haplotypes are indicated in both ACGT format and the 1–2 (major-minor allele) format. The 4-SNP haplotypes are arranged in the same order as their corresponding 3-SNP (S5-S6-S7) counterparts for the sake of easy comparison; and the 3′ most marker rs2071754 and its alleles are underlined for easy recognition. Only haplotypes with a frequency of 0.05 or above in either cases or controls are shown. The odds ratios (OR) are shown in **boldface** if their corresponding haplotypes are significantly associated with high myopia (*P*_emp_ <0.05 obtained by 50,000 permutations for each group of subjects under study). Note that the minimum *P* value achievable with 50,000 permutations is 2.00×10^−5^. The most significant result (asymptotic *P* values, Wald test; *P*_asym_) among all possible sliding windows in each group of subjects is marked by an asterisk (*). All three sets of analysis were adjusted for sex while the combined analysis was also adjusted for subject group.

One LD block identified by Haploview consisted of 7 SNPs starting from rs667773 (S2) and finishing at rs662702 (S8) (labelled as Block 1 in Figure 2). This haplotype window (indicated as S2..S8 in Table 3) gave the most significant result for sliding-window haplotype analysis among the ten possible 7-SNP sliding windows. The other two LD blocks (S9..S10 and S12..S16) gave negative results for haplotype analysis (Table 3). These results were consistent with those obtained for LD-block-based haplotype analysis by Haploview (data not shown).

### Replication study – Group 2 subjects

For Group 2 subjects, there were no significant difference in the proportions of women between cases and controls (65.2% vs 60.2%, *P*  =  0.7468; Table 1). Five SNPs were genotyped in the replication study, and their genotypes were all in HWE (Table 2). Single-marker analysis did not show any significant differences between cases and controls under all five genetic models tested (Table 2). Single-marker analysis still did not give any significant results when Groups 1 and 2 subjects were combined and analysed directly under all genetic models or when meta-analysis was conducted to compare allele frequencies for Group 1 and Group 2 subjects with control for subject groups.

To maintain consistency with haplotype analysis for Group 1 subjects, haplotype analysis was also performed for Group 2 subjects with adjustment for sex. On the other hand, allele frequencies did not differ significantly between subjects in Group 1 and in Group 2 for either cases or controls. While haplotype frequencies did not differ significantly between controls in Group 1 and in Group 2, haplotype frequencies did differ significantly between cases from these two subject groups. Therefore, haplotype analysis was carried out for Groups 1 and 2 subjects together with adjustment for both sex and subject group as covariates to avoid their potential confounding effects.

With sliding-window haplotype analysis, we successfully replicated in Group 2 subjects the significant association between high myopia and the 4-SNP haplotype window of rs2071754-rs3026393-rs1506-rs12421026 (S4-S5-S6-S7) (Table 4). Interestingly, the omnibus test was in fact slightly more significantly with 3-SNP S5-S6-S7 haplotypes (*P*_asym_ = 7.93×10^−12^) than with 4-SNP S4-S5-S6-S7 haplotypes (*P*_asym_ = 4.06×10^−11^). The direction of association was identical in both the initial study and the replication study for both S5-S6-S7 haplotypes and S4-S5-S6-S7 haplotypes (Table 4). There were two high-risk S5-S6-S7 haplotypes: TAA (112; odds ratio (OR)  = 12.7, 20.8 and 16.8 for Group 1, Group 2 and both groups combined, respectively), and GTG (221; OR = 10.3, 11.4 and 10.7 for Group 1, Group 2 and both groups combined, respectively). Similarly, there were two high-risk S4-S5-S6-S7 haplotypes: GTAA (1112; OR = 10.7, 19.4 and 15.9 for Group 1, Group 2 and both groups combined, respectively), and AGTG (2221; OR = 10.7, 13.8 and 11.8 for Group 1, Group 2 and both groups combined, respectively). On the other hand, there were two protective haplotypes. For the S5-S6-S7 window, the protective haplotypes were TAG (111; only significant in the combined group with OR = 0.767) and GTA (222; OR = 0.618, 0.592 and 0.606 for Group 1, Group 2 and both groups combined, respectively). For the S4-S5-S6-S7 window, the protective haplotypes were GTAG (1111; only significant in the combined group with OR = 0.761) and AGTA (2222; OR = 0.583, 0.600 and 0.594 for Group 1, Group 2 and both groups combined, respectively). Note that PLINK calculates OR for a particular haplotype with reference to all the other haplotypes, and hence the reference haplotypes are different for different individual haplotypes under study. It is interesting to note that the two high-risk haplotypes were each found at ∼1% in the combined control group, but at ∼9% in the combined case group (Table 4). On the contrary, the two protective haplotypes were found at ∼42–47% in the combined control group, but at ∼31–43% in the combined case group.

The SNP rs3026390 (S3) was also included in the follow-up study. According to the HapMap Chinese data, rs3026390 (S3), rs3026393 (S5) and rs12421026 (S7) are in tight LD (r^2^≥0.95) with each other and hence can act as proxies for each other. In Group 1 subjects, they were in moderate LD (r^2^≥0.62; Figure 2) with each other. Haplotypes and sub-haplotypes of these three SNPs were reported to be associated with high myopia in our previous family-based study [14]. Indeed, haplotypes and sub-haplotypes involving these three SNPs were associated with high myopia in the combined group (Table 5) with the results being essentially the same with Group 1 and Group 2 analysed separately (data not shown). The only exception was the S3–S5 window together with its sub-haplotypes, which testifies the critical importance of S7 (Table 3). The directions of association matched those shown in Table 4.

**Table 5:** Replication of *PAX6* haplotypes found to be associated with high myopia by Han et al [14].^a^

Windows	Haplotypes		Cases	Controls	OR	*P*_asym_	*P*_emp_
S3-S5-S7	Omnibus		–	–	–	2.02E-21	2.00E-05
	ATG (111)		0.4806	0.5416	0.764	0.0016	0.0118
	ATA (112)		0.1005	0.0127	10.5	4.28E-12	2.00E-05
	GGG (221)		0.0958	0.0091	15.4	6.59E-11	2.00E-05
	GGA (222)		0.3115	0.4151	0.632	2.15E-07	2.00E-05
S3-S5-0	Omnibus		–	–	–	0.1780	0.3218
0-S5-S7	Omnibus		–	–	–	1.01E-23	2.00E-05
	0TG (011)		0.4778	0.5370	0.772	0.0024	0.0157
	0TA (012)		0.1032	0.0131	10.7	1.16E-12	2.00E-05
	0GG (021)		0.1057	0.0298	3.89	6.15E-11	2.00E-05
	0GA (022)		0.3134	0.4201	0.620	9.04E-08	2.00E-05
S3-0-S7	Omnibus		–	–	–	2.04E-23	2.00E-05
	A0G (101)		0.4841	0.5559	0.741	0.0004	0.0020
	A0A (102)		0.1060	0.0225	5.02	5.91E-12	2.00E-05
	G0G (201)		0.0993	0.0109	12.3	8.41E-12	2.00E-05
	G0A (202)		0.3105	0.4107	0.643	5.72E-07	2.00E-05

a The table shows the summary of haplotype analysis for the combined group (Group 1 and Group 2 combined) with adjustment for both sex and subject group. Haplotypes and sub-haplotypes are examined for three SNPs with strong LD with each other: rs3026390 (S3), rs3026393 (S5) and rs12421026 (S7). Haplotypes are indicated in both ACGT format and the 1–2 (major-minor allele) format. For easy comparison and discussion, a zero (0) is inserted where one of the three SNPs is not included in the haplotype concerned. SNPs and their alleles are underlined for easy comparison if they form parts of the haplotypes detailed in Table 4. Results for individual haplotypes are shown only if the omnibus test is significant (i.e., *P*_emp_ <0.05 obtained by 50,000 permutations). The asymptotic *P* values (*P*_asym_) from Wald test are also indicated above.

### Subset analysis of extreme myopia cases

We performed a subset analysis on cases with SE ≤−10.0D in both eyes to explore whether any single marker was associated with extreme myopia. For Group 1 subjects, this stringent threshold reduced the number of cases down to 115 (37 males and 78 females) with a mean of 29.1 years (range, 17–48). Of the 16 SNPs tested, two SNPs showed a significant association with extreme myopia: rs12421026 (S7) was significant under all five models with best result under additive model (*P* = 0.0065), and rs11031423 (S14) was significant under additive and allelic models with best result also under additive model (*P* = 0.0277). However, neither association retained statistical significance after permutation test for multiple comparisons. We noted that rs667773 (S2) did not show any significant results for all five models tested although it was reported to be associated with extreme myopia (≤−10.0D) in one previous study [25].

For Group 2 subjects, there were 87 extreme myopia (SE ≤−10.0D) cases: 24 males and 63 females, and a mean age of 35.1 years (range, 18–51). When we performed a subset analysis for such extreme myopia cases in the replication study, all five SNPs under study showed negative results (data not shown). Combined analysis of all Group 1 and Group 2 extreme myopia cases against all controls still did not find any significant differences in allele or genotype frequencies.

## Discussion

Three linkage studies provided consistent evidence for the existence of a myopia locus on chromosome 11. A genome-wide linkage scan of 221 UK dizygotic twins first identified the *MPY7* locus (maximum LOD score of 6.1) at 40 cM on chromosome 11p13 as a myopia susceptibility locus [22]. Another study of an independent group of 485 UK dizygotic twin pairs (mean SE: −0.55±2.34D; range, −20.0D to +8.1D) demonstrated marginal evidence for this linkage [40]. Finally, a genome-wide scan of 36 white families with a mean SE of -4.0D also provided strong evidence for linkage of a myopia locus on chromosome 11 [41].

The *PAX6* gene was found to be directly below the highest peak at 11p13 and analysis of 5 tag SNPs with quantitative transmission/disequilibrium test showed strong evidence of linkage to all markers (*P* = 0.006), but no association with the SNPs or their haplotypes [22]. The refractive error of the study subjects ranged from -12.12 D to +7.25 D (mean SE = +0.39±2.38 D). In a subsequent population-based case-control study of 596 individuals from the 1958 British Birth Cohort, 25 tag SNPs from across a 530-kb region that included the *PAX6* locus and putative control regions were examined [24]. Both qualitative and quantitative trait analysis found no significant results in either individual SNPs or 3-SNP sliding-window haplotypes. The variance of refraction was 6.25 and the SD from the mean was 2.50 for these subjects although the investigators had selected subjects from the lowest and highest tertiles for case-control comparison.

The initial phase of our study recruited 600 unrelated Hong Kong Chinese subjects (Group 1) including 300 high myopia cases (SE ≤−8.00 D) and 300 controls (SE within ±1.00 D). The initial study provided convincing evidence for the association of 3′ *PAX6* haplotypes with high myopia in Chinese. In the second phase, we successfully replicated the initial positive results with another cohort (Group 2) of 299 cases and 299 controls recruited using the same criteria.

Note that the vast majority of cases of human myopia (>95%) develops due to excessive axial eye size resulting from accelerated postnatal eye growth, but not through changes in corneal or lens power [42]. In fact, our study also examined posterior axial myopia as is obvious from the strong correlation between AXL and SE in our samples.

There was no significant difference of all SNP allele frequencies in either the case group or the control group between Group 1 and Group 2 subjects. This justifies the data analysis with both groups combined wherever appropriate. The allele and genotype frequencies in the two separate control groups were also similar to those from the HapMap Chinese data (*P*>0.05; data not shown). Moreover, the LD pattern observed in the control group of the initial study was similar to that for HapMap Chinese, but slightly different from that of HapMap Caucasians (data not shown).

The initial phase of the present study captured the genetic information in a 324.6-kb region encompassing the *PAX6* locus and all potential regulatory regions by genotyping 16 SNPs. Of note was the clustering of 8 SNPs in an 8-kb region of low LD region in the 3′ end of the *PAX6* gene (Figure 1). No association was demonstrated between high myopia and each of these 16 SNPs individually under all five genetic models tested (Table 2), not even for rs3026390 (S3) and rs3026393 (S5), which were previously reported positive [14]. Without *a priori* knowledge of the haplotype window size that is most appropriate and powerful for detecting the association, we used PLINK to conduct exhaustive haplotype analysis of all possible window sizes (the variable-sized sliding-window strategy), which has been proven to be more powerful than single-marker analysis and LD-block-based haplotype analysis, particularly in regions of low LD as in the 3′ end of the *PAX6* locus [43]. The results show that, in any window size, there was at least one haplotype window significantly associated with high myopia (Table 3). The most significant association (*P*_asym_ = 3.54×10^−10^ and *P*_emp_ = 2.00×10^−5^) fell on the 4-SNP window consisting of rs2071754 (S4), rs3026393 (S5), rs1506 (S6) and rs12421026 (S7) while the second top-ranking is the 3-SNP window of S5-S6-S7 (*P*_asym_ = 5.48×10^−10^ and *P*_emp_ = 2.00×10^−5^).

In the replication study, we genotyped these 4 SNPs for Group 2 case-control subjects (Table 2). In addition, rs3026390 (S3) was also included because high myopia was found to be associated with S3 on its own and with S3-S5-S7 haplotypes in a previous study [14]. As in the initial study, these 5 SNPs did not show any significant results on their own individually (Table 2). However, the association of high myopia with the 4-SNP S4-S5-S6–S7 haplotypes and 3-SNP S5-S6-S7 haplotypes was successfully replicated in the follow-up study (Table 4). Interestingly, the results were more significant with S5-S6-S7 (*P*_asym_ = 7.93×10^−12^) window than with S4-S5-S6-S7 window (*P*_asym_ = 4.06×10^−11^). The same pattern was also observed in the combined group *P*_asym_ = 6.28×10^−23^ for S5-S6-S7 and *P*_asym_ = 1.56×10^−18^ for S4-S5-S6-S7). The results remained very significant even after correction for multiple comparisons by permutation tests. The direction of association was also consistent across both studies. In the combined group, there were two protective haplotypes and two high-risk haplotypes with odds ratios of similar magnitudes in both 3-SNP S5-S6-S7 and 4-SNP S4-S5-S6-S7 windows (Table 4). In general, protective haplotypes were more frequent than the high-risk haplotypes in the general population.

It is interesting to note that rs2071754 (S4), rs3026393 (S5), rs1506 (S6) and rs662702 (S8) were also investigated by Simpson et al [24] The study did not find any significant association for individual SNPs or 3-SNP sliding-window haplotypes S4-S5-S6 or S5-S6-S8. In our exhaustive sliding-window haplotype analysis, the S4-S5-S6 haplotype window was indeed associated with high myopia in the initial analysis (omnibus test *P*_asym_ = 0.0184), but the association did not survive correction by permutations (*P*_emp_ = 0.4799). The S5-S6-S8 haplotype window, not tested in the sliding-window approach, was found to be not associated (*P*_asym_ = 0.1060, sex-adjusted omnibus test) with high myopia on a separate analysis. While rs12421026 (S7) was found to be critically important in our study (Table 3), it was *not* studied by Simpson et al. On the other hand, rs667773 (S2) was first reported to be associated with *extreme* myopia by a Taiwanese study involving 67 cases (SE ≤−10.0D) and 85 controls [25], but did not give significant results in our initial analysis (Table 2) or a subset analysis of cases with extreme myopia (SE ≤−10.0D). Negative finding with rs667773 was also reported by another recent study examining 379 high myopia cases and 349 controls [26].

Intriguingly, three previous studies performed association study with *common* myopia and reported negative results [22]–[24], probably because of the lower heritability in less severe refractive errors [44], [45]. The difference in ethnicity could be one of the reasons for negative results. With both initial and replication testing, we confirm that 3′ *PAX6* haplotypes are associated with *high* myopia in our Chinese population. In our previous study involving 164 Chinese nuclear families with 170 highly myopic siblings, rs3026390 (S3) and rs3026393 (S5) was found to be associated with *high* myopia upon single-marker analysis [14]. The present study did not replicate these positive single markers in both the initial and the follow-up phases. However, we did successfully replicate the positive results for 2-SNP or 3-SNP haplotypes that included rs12421026 (S7) (Table 5). The consistent association of high myopia with *PAX6* haplotypes rather than single SNPs implies that the SNPs being studied are most likely not the causal variants driving the association and that the associated haplotypes are expected to carry or to be in strong LD with the causal variants.

The 4 SNPs (S4, S5, S6 and S7) of the associated haplotypes are found in the 3′ end of the *PAX6* gene and span a genomic region of ∼3.3 kb encompassing the last two exons and the 3′ untranslated region (UTR) of *PAX6*. Intriguingly, the highly conserved region of the 3′ UTR is predicted by the TargetScan programme [46] to harbour binding sites for 10 micro-RNAs (Figure 1). Micro-RNAs are small non-coding RNA molecules that bind to messenger RNAs of protein-coding genes to repress their translation and hence add an extra layer of control over gene expression [47]. It is therefore possible that variation in the *PAX6* expression level may be implicated in influencing the susceptibility to myopia development. Whether unidentified SNPs in these binding sites could be involved in driving the association remains to be determined. Of particular interest is a recent study reporting the association of high myopia with dinucleotide repeats in the *PAX6* promoter that were found to affect the transcription in a luciferase-reporter assay [26]. These studies tend to suggest that variation in *PAX6* expression may be associated with genetic predisposition to myopia development.

In conclusion, several haplotypes in the 3′ end of the *PAX6* locus were found to be associated with high myopia in both the initial study and the replication study involving Han Chinese subjects. Our study also successfully replicated the associated haplotypes found in a recent study. The study supports *PAX6* as a susceptibility gene for high myopia and provides the foundation for further investigation to identify the genuine causal variants.

## Supporting Information

Appendix S1 Detailed protocols are given for SNP genotyping, including primer information and reaction conditions.
(DOC)

## References

[1] Seet B, Wong TY, Tan DT, Saw SM, Balakrishnan V (2001). Myopia in Singapore: taking a public health approach.. Br J Ophthalmol.

[2] Vitale S, Ellwein L, Cotch MF, Ferris FL, Sperduto R (2008). Prevalence of refractive error in the United States, 1999–2004.. Arch Ophthalmol.

[3] Saw SM, Gazzard G, Shih-Yen EC, Chua WH (2005). Myopia and associated pathological complications.. Ophthalmic Physiol Opt.

[4] Tang WC, Yap MK, Yip SP (2008). A review of current approaches to identifying human genes involved in myopia.. Clin Exp Optom.

[5] Hornbeak DM, Young TL (2009). Myopia genetics: a review of current research and emerging trends.. Curr Opin Ophthalmol.

[6] Lin LL, Shih YF, Hsiao CK, Chen CJ, Lee LA (2001). Epidemiologic study of the prevalence and severity of myopia among schoolchildren in Taiwan in 2000.. J Formos Med Assoc.

[7] Edwards MH, Lam CS (2004). The epidemiology of myopia in Hong Kong.. Ann Acad Med Singapore.

[8] Kempen JH, Mitchell P, Lee KE, Tielsch JM, Broman AT (2004). The prevalence of refractive errors among adults in the United States, Western Europe, and Australia.. Arch Ophthalmol.

[9] Wu MM, Edwards MH (1999). The effect of having myopic parents: an analysis of myopia in three generations.. Optom Vis Sci.

[10] Lopes MC, Andrew T, Carbonaro F, Spector TD, Hammond CJ (2009). Estimating heritability and shared environmental effects for refractive error in twin and family studies.. Invest Ophthalmol Vis Sci.

[11] Dirani M, Shekar SN, Baird PN (2008). Adult-onset myopia: the Genes in Myopia (GEM) twin study.. Invest Ophthalmol Vis Sci.

[12] Han W, Yap MK, Wang J, Yip SP (2006). Family-based association analysis of hepatocyte growth factor (HGF) gene polymorphisms in high myopia.. Invest Ophthalmol Vis Sci.

[13] Tang WC, Yip SP, Lo KK, Ng PW, Choi PS (2007). Linkage and association of myocilin (MYOC) polymorphisms with high myopia in a Chinese population.. Mol Vis.

[14] Han W, Leung KH, Fung WY, Mak JY, Li YM (2009). Association of PAX6 polymorphisms with high myopia in Han Chinese nuclear families.. Invest Ophthalmol Vis Sci.

[15] Zha Y, Leung KH, Lo KK, Fung WY, Ng PW (2009). TGFB1 as a susceptibility gene for high myopia: a replication study with new findings.. Arch Ophthalmol.

[16] Callaerts P, Halder G, Gehring WJ (1997). PAX-6 in development and evolution.. Annu Rev Neurosci.

[17] Ashery-Padan R, Gruss P (2001). Pax6 lights-up the way for eye development.. Curr Opin Cell Biol.

[18] Tsonis PA, Fuentes EJ (2006). Focus on molecules: Pax-6, the eye master.. Exp Eye Res.

[19] Schedl A, Ross A, Lee M, Engelkamp D, Rashbass P (1996). Influence of PAX6 gene dosage on development: overexpression causes severe eye abnormalities.. Cell.

[20] Grindley JC, Davidson DR, Hill RE (1995). The role of Pax-6 in eye and nasal development.. Development.

[21] Tzoulaki I, White IM, Hanson IM (2005). PAX6 mutations: genotype-phenotype correlations.. BMC Genet.

[22] Hammond CJ, Andrew T, Mak YT, Spector TD (2004). A susceptibility locus for myopia in the normal population is linked to the PAX6 gene region on chromosome 11: a genomewide scan of dizygotic twins.. Am J Hum Genet.

[23] Mutti DO, Cooper ME, O'Brien S, Jones LA, Marazita ML (2007). Candidate gene and locus analysis of myopia.. Mol Vis.

[24] Simpson CL, Hysi P, Bhattacharya SS, Hammond CJ, Webster A (2007). The Roles of PAX6 and SOX2 in Myopia: lessons from the 1958 British Birth Cohort.. Invest Ophthalmol Vis Sci.

[25] Tsai YY, Chiang CC, Lin HJ, Lin JM, Wan L (2008). A PAX6 gene polymorphism is associated with genetic predisposition to extreme myopia.. Eye (Lond).

[26] Ng TK, Lam CY, Lam DS, Chiang SW, Tam PO (2009). AC and AG dinucleotide repeats in the PAX6 P1 promoter are associated with high myopia.. Mol Vis.

[27] Hewitt AW, Kearns LS, Jamieson RV, Williamson KA, van Heyningen V (2007). PAX6 mutations may be associated with high myopia.. Ophthalmic Genet.

[28] Fantes J, Redeker B, Breen M, Boyle S, Brown J (1995). Aniridia-associated cytogenetic rearrangements suggest that a position effect may cause the mutant phenotype.. Hum Mol Genet.

[29] Xu ZP, Saunders GF (1997). Transcriptional regulation of the human PAX6 gene promoter.. J Biol Chem.

[30] Williams SC, Altmann CR, Chow RL, Hemmati-Brivanlou A, Lang RA (1998). A highly conserved lens transcriptional control element from the Pax-6 gene.. Mech Dev.

[31] Plaza S, Saule S, Dozier C (1999). High conservation of cis-regulatory elements between quail and human for the Pax-6 gene.. Dev Genes Evol.

[32] Kammandel B, Chowdhury K, Stoykova A, Aparicio S, Brenner S (1999). Distinct cis-essential modules direct the time-space pattern of the Pax6 gene activity.. Dev Biol.

[33] Griffin C, Kleinjan DA, Doe B, van Heyningen V (2002). New 3′ elements control Pax6 expression in the developing pretectum, neural retina and olfactory region.. Mech Dev.

[34] Kleinjan DA, Bancewicz RM, Gautier P, Dahm R, Schonthaler HB (2008). Subfunctionalization of duplicated zebrafish pax6 genes by cis-regulatory divergence.. PLoS Genet.

[35] de Bakker PI, Yelensky R, Pe'er I, Gabriel SB, Daly MJ (2005). Efficiency and power in genetic association studies.. Nat Genet.

[36] Nielsen DM, Suchindran S, Smith CP (2008). Does strong linkage disequilibrium guarantee redundant association results?. Genet Epidemiol.

[37] Zhou L, Myers AN, Vandersteen JG, Wang L, Wittwer CT (2004). Closed-tube genotyping with unlabeled oligonucleotide probes and a saturating DNA dye.. Clin Chem.

[38] Purcell S, Neale B, Todd-Brown K, Thomas L, Ferreira MA (2007). PLINK: a tool set for whole-genome association and population-based linkage analyses.. Am J Hum Genet.

[39] Barrett JC, Fry B, Maller J, Daly MJ (2005). Haploview: analysis and visualization of LD and haplotype maps.. Bioinformatics.

[40] Andrew T, Maniatis N, Carbonaro F, Liew SH, Lau W (2008). Identification and replication of three novel myopia common susceptibility gene loci on chromosome 3q26 using linkage and linkage disequilibrium mapping.. PLoS Genet.

[41] Ciner E, Ibay G, Wojciechowski R, Dana D, Holmes TN (2009). Genome-wide scan of African-American and white families for linkage to myopia.. Am J Ophthalmol.

[42] Zadnik K (1997). The Glenn A. Fry Award Lecture (1995). Myopia development in childhood.. Optom Vis Sci.

[43] Guo Y, Li J, Bonham AJ, Wang Y, Deng H (2009). Gains in power for exhaustive analyses of haplotypes using variable-sized sliding window strategy: a comparison of association-mapping strategies.. Eur J Hum Genet.

[44] Guggenheim JA, Kirov G, Hodson SA (2000). The heritability of high myopia: a reanalysis of Goldschmidt's data.. J Med Genet.

[45] Farbrother JE, Kirov G, Owen MJ, Guggenheim JA (2004). Family aggregation of high myopia: estimation of the sibling recurrence risk ratio.. Invest Ophthalmol Vis Sci.

[46] Lewis BP, Burge CB, Bartel DP (2005). Conserved seed pairing, often flanked by adenosines, indicates that thousands of human genes are microRNA targets.. Cell.

[47] Bartel DP (2009). MicroRNAs: target recognition and regulatory functions.. Cell.

