# Supplementary material for: PAX6 Haplotypes Are Associated with High Myopia in Han Chinese
Source: PLoS One. 2011 May 12;6(5):e19587. doi: 10.1371/journal.pone.0019587 (PMC3093386; doi:10.1371/journal.pone.0019587)
Supplement: Appendix S1 — Detailed protocols are given for SNP genotyping, including primer information and reaction conditions. (DOC) [file pone.0019587.s001.doc]

Appendix S1

***PAX6* Haplotypes Are Associated with High Myopia in Han Chinese**

Corresponding author: Shea Ping Yip

**SNP genotyping**

For some SNPs, one allele preserves the recognition sequence for a restriction enzyme while the other allele removes the recognition sequence. Such SNPs can thus be genotyped by the method of restriction fragment length polymorphism. In this study, 10 SNPs were genotyped using restriction enzymes under conditions recommended by the manufacturer (Fermentas): rs3026354 (S1), rs3026393 (S5), rs1506 (S6), rs12421026 (S7), rs3026401 (S9), rs7125966 (S10), rs2863231 (S11), rs964112 (S12), rs7947424 (S13), and rs509628 (S16). Polymerase chain reaction (PCR) was performed in a 10-μL reaction mixture containing 10 ng of genomic DNA, 2.5 mM MgCl_2_, 0.1 or 0.3 μM each of the forward and reverse primers, 0.2 mM each dNTP and 1× PCR buffer (which contains KCl and (NH_4_)_2_SO_4_) and 0.2 U of HotStarTaq Plus DNA polymerase (Qiagen). Amplification was performed in 96-well plates with a GeneAmp 9700 PCR System (Applied Biosystems), including 1 cycle of initial denaturation for 5 minutes at 95°C, 37 cycles of 30 seconds at 95°C, 30 seconds at the annealing temperature (Table S1), and 40 seconds at 72°C, plus 1 cycle of final extension for 5 minutes at 72°C. Digested products were separated by electrophoresis in polyacrylamide gels of appropriate concentration.

The remaining six SNPs were genotyped by unlabelled probe melting analysis using a saturating dye: rs667773 (S2), rs3026390 (S3), rs2071754 (S4), rs662702 (S8), rs11031423 (S14) and rs11031419 (S15) [1]. This method uses asymmetric PCR to generate single-stranded DNA (ssDNA) product. The unlabelled probe is 3’ blocked by a phosphate group to prevent probe extension. After PCR, the unlabelled probe and a saturating dsDNA dye are added to ssDNA target for high-resolution melting analysis. Asymmetric PCR reaction was performed in a 10-μL reaction mixture containing 10 ng of genomic DNA, 2.5 or 3.5 mM MgCl_2_, 0.1-0.5 μM excess primer with a limiting:excess primer ratio of 1:5 or 1:10 (Table S1), 0.2 mM each dNTP, 1× PCR buffer and 0.2 U of HotStarTaq Plus DNA Polymerase (Qiagen). Amplification was performed in 96-well plates with a GeneAmp 9700 PCR system, including 1 cycle of initial denaturation for 5 minutes at 95°C, 50 cycles of 30 seconds at 95°C, 30 seconds at the annealing temperature (Table S1), and 25 seconds at 72°C, plus 1 cycle of final extension for 5 minutes at 72°C. After PCR, a 10-μL reaction mixture containing 8.4 μl of PCR product, 1.0 μM 3’ phosphorylated probe (synthesized by IDT or Tech Dragon Limited) and 2 μM SYTO 9 green fluorescent nucleic acid stain (Invitrogen) was prepared in 96-well white plates. Melting was performed in a LightCycler 480 instrument (Roche) with heating samples to 95°C for 1 minute and then cooling to 50°C for 1 minute. The melting data were then collected between 50°C and 95°C with a slope of 0.11°C/s at 5 acquisitions per °C, using the “melting curves” analysis mode. Samples were again cooled to 40°C for 10 seconds. All LC480 default values for rates were used: 2.2°C/s for cooling or 4.4°C/s for heating. Melting curves were analysed with LightCycler 480 Software (Version 1.5, Roche).

**REFERENCES**

1. Zhou L, Myers AN, Vandersteen JG, Wang L, Wittwer CT (2004) Closed-tube genotyping with unlabelled oligonucleotide probes and a saturating DNA dye. Clin Chem 50: 1328-1335.

**Table S1.** *PAX6* SNPs: Genotyping methods, primers, probes and key reaction conditions for PCR

| SNP ^a^ | Genotyping method | Primer sequences (5’>3’) ^b^ | Mg^2+^ (mM) | Primer (μM) | T_m_ (°C) |
| --- | --- | --- | --- | --- | --- |
| rs3026354 (S1) | RFLP (BseNI) | F: AGC TTG AGC CTG GCG AGT TT | 2.5 | 0.1 | 58 |
|  |  | R: ACC TCT CTG TCC CCA ACA CAA |  |  |  |
|  |  |  |  |  |  |
| rs667773 (S2) | UP | F: CAG TCT GCT AAA TGC TCT GC | 2.5 | 0.02 | 55 |
|  |  | R: TGG TAG ACA CTG GTG CTG AA |  | 0.1 |  |
|  |  | P: GTG CTA ACC TGT CCC ACC TGA TTT CC |  |  |  |
|  |  |  |  |  |  |
| rs3026390 (S3) | UP | F: AGT AGT TTT CTC TGA AGA GTA AAG T | 2.5 | 0.05 | 55 |
|  |  | R: CTG TGA AGA TAG CAA AAT GTG TAT |  | 0.5 |  |
|  |  | P: GTT TGA AAC AGA TTG ATT CCC CAT ACC G |  |  |  |
|  |  |  |  |  |  |
| rs2071754 (S4) | UP | F: AGG AAC TCT GCC AAC AAG TC | 2.5 | 0.03 | 55 |
|  |  | R: TAG CAG AGG TTA GAG ACA AAA ATC |  | 0.3 |  |
|  |  | P: CTA GTC CTT CCA CTG GTC ACA GCT AGA |  |  |  |
|  |  |  |  |  |  |
| rs3026393 (S5) | RFLP (MboII) | F: GCA GAC ACA CAT GAA CAG TCA G | 2.5 | 0.1 | 55 |
|  |  | R: AGA GGA GCT ATG AGG GCA AGA A |  |  |  |
|  |  |  |  |  |  |
| rs1506 (S6) | RFLP (PvuII) | F: TGG TTA CTT TCA CAC ACA CTC TG | 2.5 | 0.1 | 55 |
|  |  | R: ACT GAA GGC CTT TAA TTC CCA C |  |  |  |
|  |  |  |  |  |  |
| rs12421026 (S7) | RFLP (BseLI) | F: (T)_18_ TCT TCA CCG GCA ATT GGA CC | 2.5 | 0.3 | 55 |
|  |  | R: (T)_15_ TGT GAA CAG ATA ATT GAG ATT CCT TG |  |  |  |
|  |  |  |  |  |  |
| rs662702 (S8) | UP | F: GTC TCC AAA GTC TCT GCT GT | 2.5 | 0.03 | 56 |
|  |  | R: TGA GCA CAC ATA CGC ACT G |  | 0.3 |  |
|  |  | P: TCC CAC ATA GCC CTT CTC TGA CAG TT |  |  |  |
|  |  |  |  |  |  |
| rs3026401 (S9) | RFLP (RsaI) | F: GAG GTG GAC ATA AGG AAT AAA TGG A | 2.5 | 0.1 | 55 |
|  |  | R: (T)_18_ AGA ACA ATG CCA AAC GTA CTA TCT G |  |  |  |
|  |  |  |  |  |  |
| rs7125966 (S10) | RFLP (BseLI) | F: (T)_15_ CCA ACA GTG AAG ACA TGT GCC AAA | 2.5 | 0.1 | 55 |
|  |  | R: (T)_15_ TAC TGC GAC TCC TTA CAG CCA T |  |  |  |
|  |  |  |  |  |  |
| rs2863231 (S11) | RFLP (BseNI) | F: GAT TAG GCT GTT ATT GCA GAG ATC CAA | 2.5 | 0.1 | 55 |
|  |  | R: (T)_15_ T ACT TCA GAG TAG GTG CTA ACC CAG |  |  |  |
|  |  |  |  |  |  |
| rs964112 (S12) | RFLP by RsaI | F: CAT ACT ATG TCC TTA CTC TTG TGG | 2.5 | 0.1 | 52 |
|  |  | R: TGC ACA GCA AAT GTT CAG AAT CC |  |  |  |
|  |  |  |  |  |  |
| rs7947424 (S13) | RFLP (AluI) | F: AAA AGG CCC CAG TGT GTG TTG | 2.5 | 0.1 | 55 |
|  |  | R: TCA ATC CAT GGT CAA TCC TTA TCC |  |  |  |
|  |  |  |  |  |  |
| rs11031423 (S14) | UP | F: TGT AAT CGT GAA GGT TAC TAC CA | 2.5 | 0.03 | 56 |
|  |  | R: GCC CAA TTT CAG TTT TAA TGA GAG |  | 0.3 |  |
|  |  | P: GTA TGA GTA TCA GCC TCC CCC ATC CT |  |  |  |
|  |  |  |  |  |  |
| rs11031419 (S15) | UP | F: AAA TTA GTT GTT CTC CAC CTT GA | 3.5 | 0.02 | 56 |
|  |  | R: TTG GTT CTC TAT TAT TTT CCT GC |  | 0.1 |  |
|  |  | P: ACA TTC TAC AAG TTT TTT TCT GCT CCC A |  |  |  |
|  |  |  |  |  |  |
| rs509628 (S16) | RFLP (DraI) | F: GAC TAC TCT TCACTA ATT ATG ACA C | 2.5 | 0.1 | 52 |
|  |  | R: GTA ATT GTC TTA CTA CTG CAT GAA TTG |  |  |  |

Abbreviations: SNP, single nucleotide polymorphism; RFLP, restriction fragment length polymorphism; UP, unlabelled probe (melting analysis); and T_m_, annealing temperature for PCR.

^a^ The SNPs are listed in sequential order from the 5’ end to the 3’ end of the sense strand of the *PAX6* gene, which is the minus strand. They are also designated S1 to S16 for the sake of easy reference and discussion.

^b^ Primer and probe names are indicated before the colon. Names starting with F indicate forward primers; names with R, reverse primers; and names with P, unlabelled probes. A few primers have a poly-T tail at the 5’ end to enhance the size difference of restricted fragments for easy genotype calling. Note that all probes are phosphorylated at the 3’ end to prevent extension by DNA polymerase.
